# We don’t need more apps, we need connection: recommender systems as under-explored chance to promote students’ mental health at universities

**DOI:** 10.3389/fpsyg.2025.1629265

**Published:** 2026-01-05

**Authors:** Jennifer Apolinário-Hagen, Johannes Schobel, Anne-Kathrin Helten, Fatma Sahan, Rüdiger Pryss, Dennis John

**Affiliations:** 1 Institute for Occupational, Social and Environmental Medicine, Faculty of Medicine, Heinrich Heine University Düsseldorf and University Hospital Düsseldorf, Düsseldorf, Germany; 2 Center for Digital Medicine, Heinrich Heine University Düsseldorf, Düsseldorf, Germany; 3 DigiHealth Institute, Neu-Ulm University of Applied Sciences, Neu-Ulm, Germany; 4 Institute for Applied Research and Evaluation, Lutheran University of Applied Sciences, Nuremberg, Germany; 5 Institute of Clinical Epidemiology and Biometry, University of Würzburg, Würzburg, Germany

**Keywords:** mental health, recommender systems, digital health, student health services, health promotion, universities, ecological momentary assessment, student well-being

## Abstract

Recent research indicates that more than one-third of university students globally experience substantial stress, mild anxiety symptoms, and mild or moderate–severe depressive symptoms, respectively. Despite the clear need, existing well-established mental health services (MHS) at universities, ranging from on-site health promotion programs to mobile health apps, are rarely used by students. Barriers for uptake include on the one hand person-specific factors, such as lack of problem awareness or knowledge about available services, and on the other hand challenges related to support structures like complex registration processes, limited resources or insufficient implementation of MHS. Low-threshold, personalized approaches could improve the accessibility, acceptance, and use of existing MHS. Recognizing this mismatch between student demand and service uptake, we propose a customizable recommender system for digital and traditional MHS provided or endorsed by universities. This Perspective article proposes the concept of “ConnectedHealth@University,” a planned platform solution designed to strengthen student mental health across academic settings, and to enhance the connection, reach, and effectiveness of available university services. The proposed platform will offer stress screening, personalized recommendations, and feedback mechanisms to optimize MHS. The overarching goal is to reduce access barriers by raising awareness for study-related stressors and better aligning services with student needs. The recommender system will guide students through a range of MHS at their universities, and provide tailored recommendations based on preferences, and stress profiles. Consequently, we suggest prioritizing recommender system development and the improvement of existing interventions over creating more that may remain underutilized.

## 1 Introduction

### 1.1 Promoting mental health among students

Young adulthood is characterized by a multitude of fundamental transitions: from school to university, and then to employment. Mastering these transitions has been identified for many decades as a central developmental task of early adulthood (Arnett, 2000; Havighurst, 1972). The changes that university studies entail require significant adjustment and the development of coping strategies for the associated psychosocial stressors (Skinner et al., 2003). Time pressure due to deadlines and worries concerning exams are well-known risk factors for mental disorders among students, particularly depression and anxiety disorders, which are often associated with reduced motivation and academic performance (Duffy et al., 2020).

Recent studies report increasing stress and mental health issues among students (Heumann et al., 2024; Li et al., 2022; Tsiouris et al., 2023; Vidović et al., 2024). In an umbrella review of 62 meta-analysis with more than 8.7 million participants, Paiva et al. (2025) found relatively high prevalence rates for symptoms of mental disorders among university students, especially for depressive symptoms (mild: 35.41%, moderate: 24.54%, severe: 13.42%), anxiety (mild: 40.21%, moderate: 28.18%, severe: 16.78%), sleep disorders (mild: 41.09%, moderate: 23.3%, severe: 13.02%), and increased stress prevalence of 36.34%. In a survey of 24,533 students at 13 German universities, approximately 40–50% reported (almost) always or frequently feeling exhausted during their daily studies or experiencing burnout symptoms during the COVID-19 pandemic (Heinrichs et al., 2024). The changes in study conditions in many degree programs brought about by the COVID-19 pandemic (e.g., decrease in social contacts) may also be associated with increased rates of depressive symptoms especially in countries with higher income (Paiva et al., 2025), and could have a lasting impact on the mental health and qualifications of the next generation of academics, who are so urgently needed to build crisis-resilient societal structures.

However, according to international research, students with mental health needs show poor help-seeking activities, especially male students (Pei et al., 2024). At the same time, female undergraduate students have a higher likelihood of experiencing mental health issues (Kartikasari et al., 2025). These findings indicate that existing mental health services (MHS) fail to reach students comprehensively; instead, there is a demand for tailored interventions that are actually used, especially before the manifestation of symptoms requiring treatment.

### 1.2 Fragmented mental health services at universities

Universities are central to student health promotion, encompassing both setting-based and behavioral prevention. The World Health Organization (WHO) has recognized the mismatch of mental health issues and actual uptake of MHS among university students several years ago (Cuijpers et al., 2019). Numerous universities have also acknowledged the urgent need to support students’ developmental tasks and have launched (more or less mandatory) nation-wide initiatives to promote students’ mental health around the world, e.g., in China (Yu et al., 2025), India (Jaisoorya et al., 2023) or Canada (Read et al., 2023). In contrast to voluntary programs and frameworks for promoting students’ mental health in countries like the United States (Rolin and Appelbaum, 2025) or Australia (Hutchesson et al., 2025; Luu, 2025), German policy makers have legally anchored this duty in the Prevention Act (§20 Social Code Book V), enshrining the funding of health-promoting programs on the expense of statutory health insurance companies for the vast majority of citizens, including university students (Hungerland et al., 2022). Universities typically offer or suggest varied psychological services, ranging from counseling centers to referrals to psychotherapeutic outpatient clinics with individual consultations. For instance, psychological counseling, available on-site and online, is a common and beneficial service (Cerolini et al., 2023; Pizzo et al., 2024), yet varies significantly by country, location, legal status, and institutional size (Franzoi et al., 2022). Although high-income countries typically offer a wide range of on-campus MHS, these resources are often underutilized, disjointed, or poorly coordinated, highlighting a key international challenge (Osborn et al., 2022).

### 1.3 Accessing suitable mental health services

Despite the high prevalence of mental illness among students (Heumann et al., 2024; Paiva et al., 2025), university MHS are underutilized, especially among vulnerable student groups (Hyseni Duraku et al., 2024). A systematic review (Zhao et al., 2025) found that only 28% of students with mental health issues actively sought help, though 41% intended to.

Barriers to seeking help are both individual and institutional. At the individual level, key obstacles include self-reliance, lack of time, limited awareness of resources, under-recognition of early symptoms, and the belief that they do not need formal support (Zhao et al., 2025; Ebert et al., 2019; Krümmel et al., 2023; MacDonald et al., 2022). At the institutional level, fragmented responsibilities, regulatory obstacles and limited resources further hinder comprehensive psychosocial support (Hyseni Duraku et al., 2024; Priestley et al., 2022; Roy et al., 2025).

To extend on-campus MSH, such as stress management, mindfulness and relaxation courses, flexible low-threshold digital MHS have also been increasingly developed, showing efficacy in reducing stress and depressive symptoms among university students (Harrer et al., 2018; Harrer et al., 2021; Matos Fialho et al., 2025). The initial barrier, though, is students recognizing their need for support and the drivers for utilization for long-term preventative effects (Krümmel et al., 2023). Subsequently, they must actively register for these MHS, a process particularly challenging for first-year and international students unfamiliar with the diverse range of university support providers (Schweighart et al., 2024).

Additionally, despite the initial optimism surrounding pilot programs, the sustainable integration of digital MHS into university structures remains limited (D’Adamo et al., 2023), and students lack information regarding trustworthy digital MHS (Montagni et al., 2020; Vomhof et al., 2024). Furthermore, universities often lack financial and personnel resources for the permanent implementation of quality-approved digital MHS, and costs for implementation as well as maintenance of digital MHS for university students are seldom reported (Taylor et al., 2024). Integrating mental health promotion and education on self-help digital MHS into curricula (e.g., Car et al., 2025; Sahan et al., 2024; Till et al., 2024) is promising, but oftentimes restricted to specific subjects (e.g., medicine). Further established and implementable solutions exist to improve service awareness and uptake, such as offering app-based recommender systems, including stress screening and feedback tools, such as “TrackYourStress” (Pryss et al., 2019), which provide individualized service recommendations and facilitate registration and initial engagement.

## 2 Health recommender systems as a promising strategy

Health recommender systems (HRS) help users find relevant services and information by reducing choice overload and make better choices aligning with personal needs and preferences, which could improve motivation, engagement with MHS and support behavior change (De Croon et al., 2021; Valentine et al., 2023). Research on HRS shows promise in increasing the uptake and engagement with digital MHS, such as apps for anxiety and depression (Cheung et al., 2018).

Ethical challenges involve lack of explainability, privacy-personalization trade-offs, and control over app usage data (Valentine et al., 2023). Useful mental HRS require clear standards, secure data handling, and ongoing refinement based on user feedback (Tapuria et al., 2024). Machine Learning (ML), including rule-based mechanisms for assessment and content tailoring, is commonly used to personalize MHS for young people, with Large Language Models and generative Artificial Intelligence gaining recent attention (Wanniarachchi et al., 2025). Despite their promises, HRS still face challenges such as bias and overfitting (Slade et al., 2024). Bias can occur at different stages (e.g., conception, data collection) and from several sources, including human (e.g., confirmation bias), data (e.g., sampling, selection, participation bias), and algorithms (Hasanzadeh et al., 2025). Hence, during the development and implementation of HRS, both representative samples as well as insights from formative qualitative research are crucial to address diverse sources of bias (John et al., 2016). This includes needs assessment as well as inquiries on information preferences of students regarding (digital) MHS (Vomhof et al., 2024; Braun et al., 2023). Key organizational stakeholders should also be involved in each stage, especially student counseling centers as oftentimes first and main referral source (D’Adamo et al., 2023).

Various approaches for tailoring and providing recommendations already exist. A scoping review by Cheung et al. (2019) demonstrated a broad heterogeneity in applied HRS approaches, including content-based, collaborative, and hybrid filtering (combining methods), with recommendations often delivered via in-app messages. Combining collaborative methods with demographic and knowledge-based filtering can enhance user experience in demand-tailored digital, on-site and blended health programs. Such hybrid HRS show promise in advancing personalized digital health solutions (Cheung et al., 2019).

Cai et al. (2022) reviewed 63 studies across 24 health domains and found that knowledge-based algorithms were most common but noted limited research and called for more dynamic user modeling, open-source knowledge bases, and large-scale evaluations.

These selected findings on the HRS research landscape provide guidance and valuable insights into the general strategies as well as the chances and risks of such platforms, which can be well applied to the development of a data-driven mental HRS for university students.

In this Perspective article, we thus present a concept for such a planned HRS. Given the early stage of development, the following illustration is not grounded on empirical data.

## 3 Illustrating objectives and features of a digital screening and recommender platform

As a promising example of a low-barrier digital platform with screening tools and an integrated recommender system, we present our ongoing vision for “ConnectedHealth@University.” In this context, we consulted key stakeholders from different organizations (e.g., psychological counseling centers, student representatives, student unions, health insurance companies) to assess key features and important requirements. These requirements will be refined in the next steps of this project and further elaborated in workshops with selected participants fostering co-creation approaches.

This concept for a digital platform intends to address the prevention paradox of high student demand for mental health support alongside the underutilization of existing university health promotion services. To achieve this, “ConnectedHealth@University” involves the following goals:

- ***Goal 1***: Raising awareness by offering low-threshold onboarding and everyday screening options for students in the form of self-tests (e.g., perceived stress related to exams or time pressure). This may help increase awareness of students’ own stress and strain profiles, especially when combined with psychoeducation and fact sheets on mental health promotion.
- ***Goal 2***: Matching services with students’ needs by providing demand-tailored suggestions via a recommender system. Personalized recommendations will take existing digital, on-site, and blended MHS at the university, as well as associated external digital MHS, into account based on students’ individual stress profiles and stated preferences. The recommender system is intended to identify and integrate existing MHS and related data, aiming for a seamless interface and low thresholds for registration and service utilization (e.g., through an integrated search form).
- ***Goal 3***: Understanding and improving services and their uptake by establishing a feedback and monitoring system to assess satisfaction and acceptance (e.g., perceived usefulness and relevance of recommendations), as well as the intended and actual use of MHS at the respective university.

## 4 Scientific and technical objectives of the digital platform

The platform will be designed to help students better understand their stress triggers and psychosocial resources in their academic setting, while also facilitating access to existing MHS at participating universities. The overall goal of the platform’s concept is to offer easily accessible referrals and connections between university support services to mitigate the negative impacts of mental health issues on students and to enhance their academic functioning.

### 4.1 Technical requirements

From a technical perspective, the platform will fully leverage state-of-the-art technologies to maximize user acceptance, while providing transparency by adhering to reporting guidelines for HRS (De Croon et al., 2021). Beyond the already mentioned participatory development approach, we identify the following technical features as essential, as shown in Table 1.

**Table 1:** Technical features of the proposed “ConnectedHealth@University” platform.

Feature	Description
Persuasive design	Application of persuasive design principles to optimize user experience while explicitly avoiding dark patterns.
Federated identity management	Support for Single-Sign-On mechanisms to enable an easy-to-use, established, and GDPR-compliant authentication process at universities, without linking system metrics and academic records (data minimization and transparency).
Gamification elements	Incorporation of gamification elements (e.g., badges, achievement systems) to enhance user engagement and long-term motivation, while considering user needs and limitations (Cheng and Ebrahimi, 2023).
Configurable EMA settings	Configurable EMA options allow users to personalize scheduling and frequency of prompts for stress ratings (Weber et al., 2022).
Time-series visualizations	Visualizations of individual metrics and characteristics enable users to track trends and progress over time (Schleicher et al., 2023).
Data linkage capabilities	Facilitate seamless connections with related support services (e.g., counseling for students with chronic illness) to ensure timely referrals and interventions.
Smartphone sensor data (optional)	On-demand use of smartphone sensor data (e.g., location, accelerometer) to infer behavioral patterns, such as decreased physical activity, as potential indicators of mental-health changes.
Multi-stage validation of algorithms	Validation of recommender algorithms via offline evaluation (historical data, e.g., “TrackYourStress”), web-based A/B testing, and user-centered focus groups (feedback on usefulness, trustworthiness, cultural fit, and relevance). On the long run: psychological outcome assessment through randomized controlled trials (e.g., control group uses MHS without prior tailoring via recommendations).
Algorithm benchmarking	Comparison of recommender algorithms using benchmark datasets (comparing filtering methods, based on the same EMA data) and web-based trials with students (random allocation to filtering methods to examine effects on engagement and satisfaction).
Continuous monitoring	Continuous monitoring after deployment through engagement dashboards (e.g., given and followed recommendations, clicks), fairness and bias testing (e.g., counterfactual fairness; Gao et al., 2025), and safety monitoring (SOP with automated rule-based triggers based on expert advice for elevated stress levels).
GDPR compliance and data protection	All data stored within the database is encrypted by default, as collected information may contain sensitive information of students. To separate identifying information, such as user profile information and login credentials, from health-related information (collected via mobile apps), two separate databases should be used. This approach is common in complex healthcare related scenarios, which is also described in the German Medical Informatics Initiative (MII; Erdfelder et al., 2023).

EMA, Ecological Momentary Assessment; GDPR, General Data Protection Regulation; SOP, Standard Operating Procedure.

To support these capabilities, modern cross-platform architecture will be implemented, consisting of a web application (fully responsive for mobile devices) and native mobile apps for Android and iOS that can be distributed via official stores. The architecture of the sensor framework is detailed elsewhere (Karthan et al., 2022). Ideally, the apps should also connect with relevant external stakeholders (e.g., health insurers, counseling centers) to ensure real-time access to updated content.

In order to ensure a high privacy and security standard, we intend to clearly separate Identity Data (IDAT) from Medical Data (MDAT), which is a common approach in medical informatics (Lablans et al., 2015; Spitzer et al., 2009). Login information, including the e-mail address containing students’ names and profile data, will be stored within the IDAT database, while all collected information and recommendations are stored in a separate MDAT database. Depending on the system, there may be two different databases (i.e., like two different relational databases), two different files (i.e., like two different SQLite databases), or one large database with two different schemas (i.e., PostgreSQL). Most importantly, this approach guarantees that no identifying information is stored within the MDAT database. Additionally, both databases should be encrypted by default (with different keys).

To enhance students’ acceptance, a mixed-methods, co-creation approach will be applied. In a first step, interviews with experts from respective domains are conducted to assess basic requirements of the platform (e.g., instruments, features of the recommender system, etc.). Next, a comprehensive user-interface will be developed and evaluated with target end users to meet their demands. Feedback will be collected via an online survey and incorporated in the apps via rapid-development cycles (e.g., using the System Usability Scale; Bangor et al., 2008; Hyzy et al., 2022).

Initially, the recommender system balancing personalization and privacy will mainly process user-entered data on needs (e.g., Ecological Momentary Assessment; EMA) and stated preferences for hybrid filtering of suitable MHS (using content- and knowledge-based, see Table 2). The acceptance of collaborative filtering and more sensitive data collection required for context-aware filtering will be investigated using participatory research approaches prior to possible implementation.

**Table 2:** Stepwise application of filtering approaches for personalizing recommendations to students.

Layer/method	Chances	Challenges/risks	Exemplary use case
On-boarding	Stress screening plus feedback for increasing awareness and initial tips	Generic advice can impede the initial motivation to engage, response burden	Explanation of screening results and first tips → fact sheets: stress coping, on-campus MHS
Hybrid filtering (Content- + Knowledge-based Filtering)	Safe, explainable recommendations based on EMA and preferences; supports early engagement	Risk of repetitive suggestions over time (based on cut-off scores, usefulness ratings and stable preferences, if-then expert rules)	Exam stress (needs identified by EMA) → stress management courses (preferences: on campus vs. online, schedule)
Context-aware filtering (with option to opt-out)	Just-in-time advice; aligns with daily rhythms, study schedules, sensor data	Privacy-sensitive; requires contextual data (time, location, wearables)	Night-time rumination/test anxiety → progressive muscle relaxation and mindfulness resources (e.g., audio files)
Collaborative filtering (with option to opt-out)	Strategies/MHS endorsed and/or used by peers may increase credibility and engagement, and help explore new MHS	Requires larger (representative) samples/data sets to capture diversity; risk of increasing bias (e.g., filter bubbles, popularity bias)	Procrastination → students with comparable background (e.g., stress levels, study subject), “found this time management workshop helpful”
Federated learning (across institutions)	Cross-university recommendation model (using aggregated data, privacy-preserving, enhanced generalizability and accuracy), greater portfolio of resources (e.g., digital MHS shared or varying institutions and cultures)	Technically complex; reduced explainability, limited by heterogeneity and diversity (e.g., EMA measures, sampling bias), cross-university governance	Self-guided online stress management courses shared across universities based on stress profiles (see StudiCare; Harrer et al., 2021)

EMA, Ecological Momentary Assessment; MHS, Mental Health Services. Exemplary use cases are provided for illustration.

Before using the app, students will provide informed consent, clearly understanding how and why their data is used and making an informed decision about the personalization-privacy trade-off. Our recommender system will initially use a hybrid approach (content- and knowledge-based filtering), which is safe and explainable, matching students’ EMA responses and stated preferences with relevant MHS. While this can become repetitive, context-aware filtering can provide just-in-time suggestions if students agree to share more sensitive data (e.g., sensor data). We recognize that collaborative filtering offers novel, peer-endorsed suggestions but requires a large dataset and risks exacerbating bias (Stinson, 2022). Finally, we propose federated learning (FL) for privacy-preserving model training directly on user devices. FL will improve cross-institutional generalizability, enhance trust in personalized MHS suggestions across multiple universities (using the same EMA instruments), and enable larger-scale performance, potentially in an international context. Nonetheless, FL approaches are still at an early stage (Grataloup and Kurpicz-Briki, 2024).

We plan to evaluate the system through a controlled field study (e.g., A/B trial) to assess its effectiveness regarding changes in students’ mental health outcomes based on the specific MHS it recommends. In addition, we will employ a mixed-methods adoption assessment, combining usage analytics with online surveys and qualitative interviews to understand user acceptance, perceived usefulness, and factors influencing sustained engagement as well as practical applicability (see Table 1). A concrete study design must be discussed with stakeholders from different domains in order to gain broader insights.

### 4.2 Content components and modules of the platform

**Figure 1:**
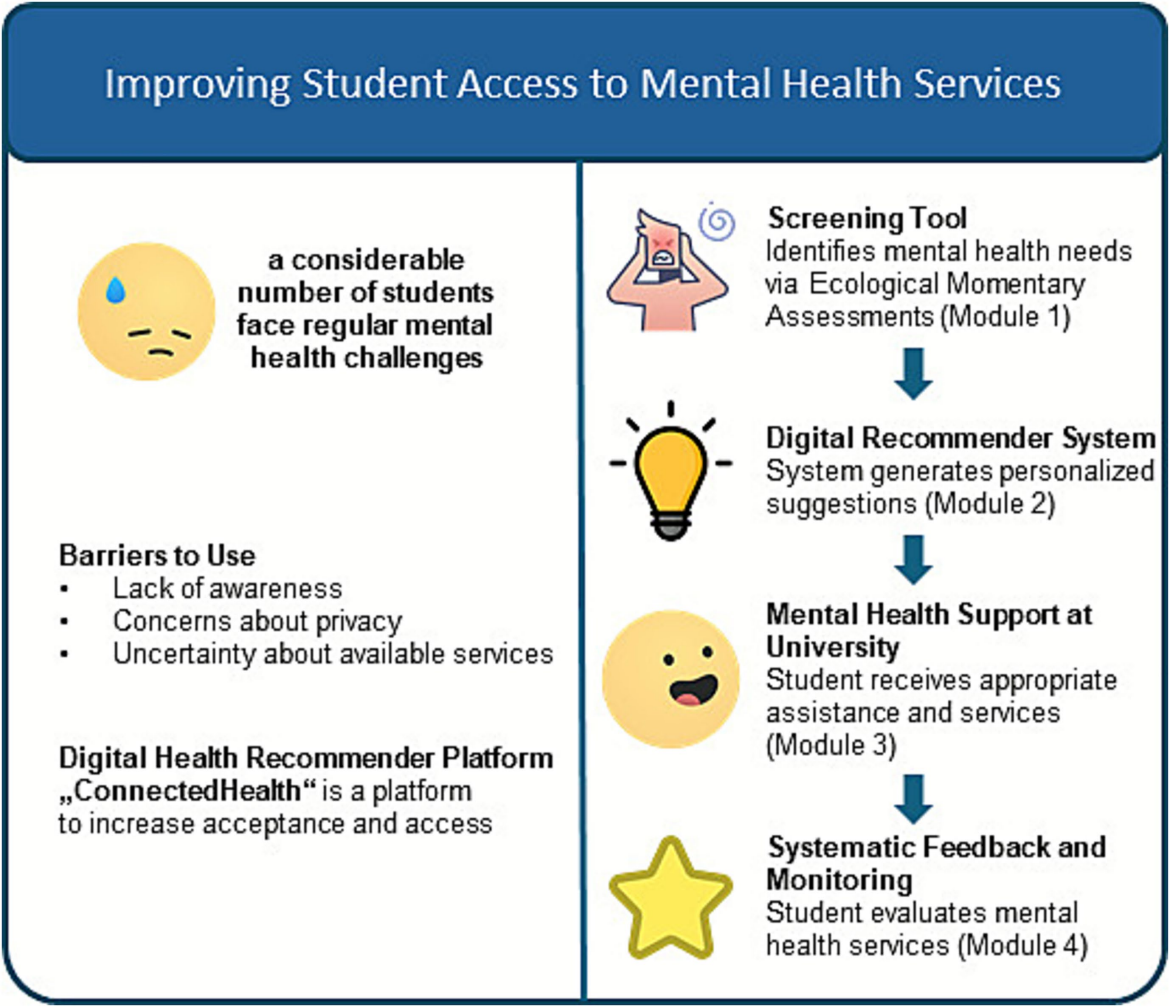
Content components of the platform “ConnectedHealth@University” in response to common barriers to use of mental health services among university students. This figure illustrates the goals and main features of a unified university platform for student mental health promotion, outlining common barriers and how recommender systems can mitigate them based on the modular structure of the proposed platform “ConnectedHealth@University” (self-created illustration).

From a content perspective, the platform concept includes the following modules (see Figure 1).

**Module 1: “I can learn more about myself and my health.”**

The screening tool is based on EMA (Shiffman et al., 2008) that can be augmented with a mobile crowdsensing solution (Pryss et al., 2018). Data gathered through crowdsensing can be used to identify university-specific stress scenarios in larger student groups, such as exam situations and the possibility to provide feedback to students (Pryss et al., 2019; Pryss et al., 2019). Students have access to various screening tools (e.g., subjective stress levels, dealing with test anxiety, or time management problems) and receive individualized feedback on their mental health status. Through continuous use of such a digital platform via a mobile app in everyday life, students are provided with on-time feedback on their stress levels and stress-inducing situations in their study routines. The screening can be complemented with psychoeducation and brief exercises. Such immediate support is important, as students experiencing acute stress (e.g., due to exams) may prioritize targeted short-term relief on long-term preventive measures (Krümmel et al., 2023).

**Module 2: “I can use suitable health services at my university.”**

Based on the results of the screening tool (see Module 1), an individualized recommendation of support services can be made at the respective local university as a trustworthy information source (Vomhof et al., 2024; Braun et al., 2023). This module acts as a kind of guide, selecting relevant MHS from a stored database and presenting demand-tailored suggestions to the students (e.g., sorted by suitability or personal relevance). The platform may contain a uniform registration form for the federated authentication for respective events or services, so that students can register directly. This eliminates several access barriers, such as different registration forms or information channels. Module 2 aims to facilitate access to existing MHS through comprehensive information and low-threshold registration options (Apolinário-Hagen et al., 2023).

**Module 3: “I can participate in a digital health service.”**

To increase the reach of health promotion, many universities already provide their students free access to a range of digital mental health resources, such as “TrackYourStress” (Pryss et al., 2019; O’Rourke et al., 2022). Some MHS have been developed in-house by universities. Others are offered free of charge to students in cooperation with partners, such as health insurance companies. Besides on-site interventions, many students are unaware of available digital MHS (Schweighart et al., 2024; Apolinário-Hagen et al., 2021). Likewise, the overly wide range of registration options for digital MHS represents a key barrier to access and uptake among students (Dederichs et al., 2021) as well as perceived lack of personalization in digital MHS (Riboldi et al., 2024). The goal of the “ConnectedHealth@University” platform is to provide personalized guidance on digital MHS, such as “StudiCare Stress” (Harrer et al., 2021). This includes direct download and registration options for digital MHS to enable low-threshold access (see Supplementary Figure S1).

**Module 4: “I can evaluate the health services offered by my university.”**

Although methods for measuring user satisfaction and acceptance of MHS are well-described, systematic collection of feedback data is rarely used at universities, especially by student counseling centers that often have limited personal resources as well as no access to suitable digital documentation systems (Farahani et al., 2023). In addition, there is scarce systematic research on the actual utilization rates of these MHS for students, with globally largely varying rates (Osborn et al., 2022).

The platform “ConnectedHealth@University” incorporates a module to establish consistent and continuous feedback data on MHS and a satisfaction monitoring system for existing MHS. To achieve this, the platform regularly sends notifications, asking students to rate respective programs. The feedback results may be shared with relevant stakeholders at each university and through networks. These reports are anonymized and provided in compliance with data protection regulations such as GDPR for European settings, using a user-friendly dashboard solution.

## 5 Discussion

Research consistently shows a significant gap between university students’ mental health needs and their help-seeking behavior, influenced by personal factors such as low problem awareness and systemic barriers across many universities globally (Zhao et al., 2025). Especially student-based barriers seeking mental health support such as the preference for self-reliance could be addressed by tailored digital solutions (Ebert et al., 2019).

Hence, more efficient mental health promotion strategies are needed for university students, ideally starting in freshmen week before study-related stressors emerge (Dederichs et al., 2021). In Canada, Velmovitsky et al. (2025) trained ML models using survey data from undergraduate students to predict risks of common mental health issues, which aims to form the basis for a system offering personalized recommendations for MHS.

While recommender systems represent a promising avenue to support student mental health, further empirical research is needed to evaluate their effectiveness. At the same time, successful implementation requires the involvement of key stakeholders, particularly in developing and applying guidelines for selected mental health apps (Khan et al., 2023). Moreover, providing students with clear information and direct registration options has been shown to be vital for increasing the uptake of digital MHS (Harrer et al., 2021; Apolinário-Hagen et al., 2023).

Considering the outlined substantial gaps, the “ConnectedHealth@University” concept centers on the stakeholder-driven development and implementation of digital platforms that integrate HRS to precisely match students’ needs with suitable existing university support services. Digital screening and feedback functions enable tailored recommendations based on individual preferences for format (digital, in-person, blended) and intervention framework (e.g., duration, individual versus group setting). Student feedback on service acceptance and satisfaction will help optimize and expand present health promotion measures.

### 5.1 Implementation challenges and strategies

Ethical challenges, including fairness and equity, must be considered throughout the development of recommender systems, especially in FL (Grataloup and Kurpicz-Briki, 2024). Self-selection bias is a common challenge, as current digital MHS research predominantly relies on female and White participants (Taylor et al., 2024). Students who participate are often those already informed about support, and thus may not represent the primary target group. To avoid this, recruitment strategies for co-creation must be adapted (Hasanzadeh et al., 2025). We will gather historical data from diverse student subgroups using the established “TrackYourStress” app (Pryss et al., 2019; O’Rourke et al., 2022) that applies validated EMA measures. This will be complemented by larger representative survey data for the platform’s “cold start,” as recommended (Hasanzadeh et al., 2025; Stinson, 2022). Our project will start with at least four different universities across Germany.

Personalization based on sociodemographic characteristics such as ethnicity will be avoided to prevent oversimplification and prejudice perpetuation (Taylor et al., 2024). Instead, personalization will use assessed needs (via EMA) and stated preferences. The acceptance of further features (context-aware filtering, preferred authentication) will be explored in formative qualitative research (e.g., focus group discussions). Since EMA and other health data are sensitive under GDPR (Ienca and Malgieri, 2022; Zhang et al., 2025), we will prioritize transparency and user control. Students will be fully informed about data use with a clear opt-out option. Because the platform targets health promotion and early intervention, not acute crises, a minimal safety protocol will be implemented, automatically triggered by elevated stress scores to provide immediate, generic, expert/rule-based information on regular support services. The platform will neither include questions about suicidal ideation or clinical screening, nor provide direct access to healthcare. Students will be informed that the system is not a substitute for therapist contact or suitable for severe issues.

Sustainable platform implementation requires context-sensitive, early involvement of key stakeholders (Cross et al., 2025), such as on-campus psychological counseling centers as gatekeepers, and practical guidance for university staff on updating MHS in the platform. Staff buy-in is thus crucial, necessitating measures like participatory approaches to increase commitment, like communicating benefits (e.g., reduced workload for general advice), and providing training and support. In addition, the platform must be integrated into existing IT infrastructure, including digital support systems of counseling centers. Ongoing maintenance should be ensured through the platform’s design (reliable, safe personalized recommendations via FL; shift to passive data collection/tracking; reduced effort for students and staff) and long-term implementation strategies, including funding, workflow integration and training (Berardi et al., 2024; Löchner et al., 2025; Nair et al., 2024). Both institution-wide and cross-university governance must be considered.

German universities benefit from the Prevention Act (Hungerland et al., 2022) for establishing cross-institutional MHS recommender systems. A realistic approach involves using competitive funding schemes for initial piloting and validation, while engaging health insurance representatives early to secure future implementation.

## 6 Conclusion

By sharing the concept of the “ConnectedHealth@University” platform, we aim to present a blueprint for a platform that can help overcome multiple barriers preventing university students from using existing MHS throughout their academic journey.

Specifically, by revealing underexplored patterns of actual use, we expect the platform to support a more effective alignment of MHS with students’ needs.

We, an interdisciplinary team of researchers specializing in the user-centered implementation of (digital) health services, call for a shift in priorities: We encourage researchers, funders, and stakeholders at universities to comprehensively employ the potential of HRS for low-threshold, tailored student mental health promotion. This approach involves shifting focus toward connecting and refining existing interventions before primarily investing further efforts and resources in (generic) novel ones that often fail to overcome common individual and structural barriers to sustainable real-world implementation.

## Data Availability

The original contributions presented in the study are included in the article/Supplementary material, further inquiries can be directed to the corresponding author.
