# Supplementary material for: We don’t need more apps, we need connection: recommender systems as under-explored chance to promote students’ mental health at universities
Source: Front Psychol. 2026 Jan 5;16:1629265. doi: 10.3389/fpsyg.2025.1629265 (PMC12813139; doi:10.3389/fpsyg.2025.1629265)
Supplement: Supplementary file 1 [file Supplementary_file_1.pdf]

Supplementary Material

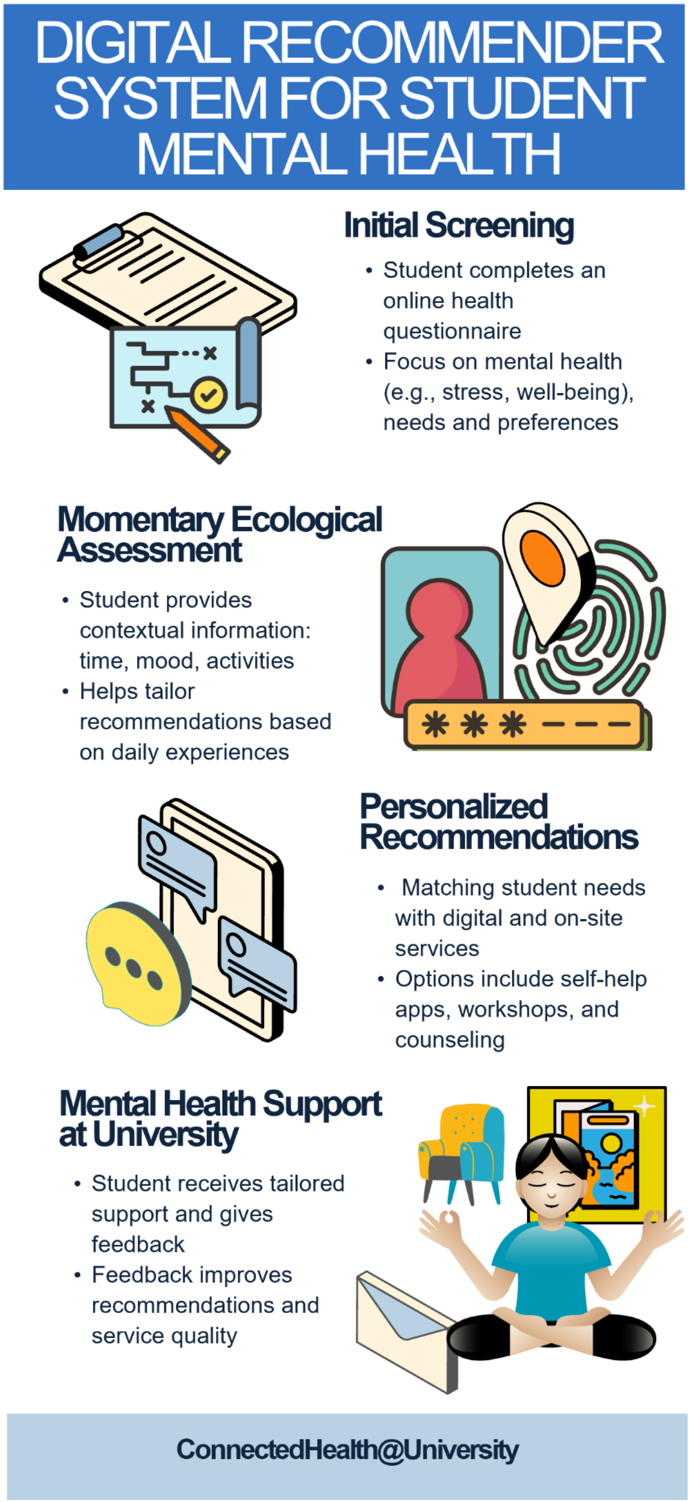

FIGURE S1
Key steps of using the digital recommender system: simplified illustration of the pathway from screening to getting support via the "ConnectedHealth@University" platform. This figure shows an example for a poster design adapted to the audience of university students (self-created using a Canva template).

Source

Perspective Article:

Apolinário-Hagen J, Schobel J, Helten A-K, Sahan F, Pryss R and John D (2025) We don't need more apps, we need connection: recommender systems as under-explored chance to promote students' mental health at universities. *Front. Psychol.* 16:1629265. doi: 10.3389/fpsyg.2025.1629265
